# Enhanced piezoelectric properties of vertically aligned single-crystalline NKN nano-rod arrays

**DOI:** 10.1038/srep10151

**Published:** 2015-05-08

**Authors:** Min-Gyu Kang, Seung-Min Oh, Woo-Suk Jung, Hi Gyu Moon, Seung-Hyub Baek, Sahn Nahm, Seok-Jin Yoon, Chong-Yun Kang

**Affiliations:** 1 Electronic Materials Research Center, Korea Institute of Science and Technology, 39-1, Hawolgok-dong, Seongbuk gu, Seoul, 136-791, Republic of Korea; 2 KU-KIST Graduate School of Converging Science and Technology, Korea University, 145, Anam-ro, Seongbuk gu, Seoul, 136-701, Republic of Korea; 3 Department of Materials Science and Engineering, Korea University, 145, Anam-ro, Sungbuk gu, Seoul 136-701, Republic of Korea

## Abstract

Piezoelectric materials capable of converting between mechanical and electrical energy have a great range of potential applications in micro- and nano-scale smart devices; however, their performance tends to be greatly degraded when reduced to a thin film due to the large clamping force by the substrate and surrounding materials. Herein, we report an effective method for synthesizing isolated piezoelectric nano-materials as means to relax the clamping force and recover original piezoelectric properties of the materials. Using this, environmentally friendly single-crystalline Na_x_K_1-x_NbO_3_ (NKN) piezoelectric nano-rod arrays were successfully synthesized by conventional pulsed-laser deposition and demonstrated to have a remarkably enhanced piezoelectric performance. The shape of the nano-structure was also found to be easily manipulated by varying the energy conditions of the physical vapor. We anticipate that this work will provide a way to produce piezoelectric micro- and nano-devices suitable for practical application, and in doing so, open a new path for the development of complex metal-oxide nano-structures.

The piezoelectric effect is the key active phenomena in microelectronic devices that convert electrical energy into mechanical energy, or generate electrical energy from mechanical energy. This effect, however, tends to be degraded when materials are synthesized as a thin film, as the electro-mechanical movement and induced external mechanical strain becomes more highly dependent on the substrate used. Since the strong clamping force generated by a rigid substrate and surrounding materials disturbs both the mechano-electrical and electro-mechanical transformation, the development of free standing or isolated piezoelectric materials represents the most effective route to enhancing the performance of micro- and nano-sized piezoelectric devices^1^,^2^,^3^,^4^,^5^,^6^,^7^,^8^. Moreover, the greater piezoelectricity offered by such materials can also provide practical functionality for devices such as piezoelectric energy harvesters^9^,^10^,^11^,^12^,^13^,^14^, sensors^15^,^16^, etc. To this end, we herein demonstrate a relaxation of substrate clamping induced piezoelectricity degradation through the successful isolation of nano-sized Na_x_K_1-x_NbO_3_ (NKN) piezoelectric materials from their surroundings. This is achieved by controlling the energy conditions of pulsed laser deposition (PLD) to control the growth and final shape of NKN nano-rod arrays.

Although the high piezoelectric coefficient and electromechanical conversion efficiency of nano-structured single-crystals of perovskite oxides such as PbZr_1-x_Ti_x_O_3_ (PZT)^17^,^18^ and (1-x)Pb(Mg_1/3_Nb_2/3_)O_3_-xPbTiO_3_ (PMN-PT)^9^,^19^ makes them ideally suited for use in piezoelectric devices, their reliance on Pb conflicts with strict health and environmental guidelines such as the Restriction of Hazardous Substances Directive (RoHS). However, the discovery by Saito et al. of high piezoelectricity in NKN ceramics^20^ has provided an alternative to Pb-based materials that is both non-toxic and environmentally friendly. Up to now, the piezoelectric properties of NKN based ceramics have been steadily improved, but the high volatility of alkali elements means that the synthesis of fine NKN ceramics is still quite difficult^21^. In recent reports, perovskite oxide based nano-materials have been produced relatively easily through a hydrothermal chemical route^9^,^12^,^16^,^18^,^22^,^23^, but achieving a nano-structured complex oxide by such methods requires either toxic metal organic precursors with reactive metal catalysts, nano-structured templates, or seed crystals. Moreover, it is difficult to control the morphology and growth direction of the resulting nano-structure, and the stoichiometry of the complex metal oxide. Accordingly, there have been only very few papers pertaining to the chemical synthesis of single-crystalline nano-structured NKN^24^,^25^,^26^, with these invariably only producing a powder form that greatly limits the control over the nano-structure of micro- and nano-sized piezoelectric devices.

More recently, Chen et al. demonstrated the physical growth of taper-like PZT nanowire arrays by PLD^27^, a method which has previously allowed for the creation of high-quality thin films of single-crystalline perovskite oxides with well-regulated stoichiometry. In adapting this method in this study to the synthesis of well-organized single crystalline NKN nano-structures, it was hoped to significantly improve their piezoelectric properties. The effect of the growth mechanism and final shape of the NKN nano-structure was also investigated by varying the lattice strain induced interfacial free energy and physical vapor conditions.

A schematic of the conventional pulsed laser deposition setup used for the physical vapor synthesis of piezoelectric NKN nano-rods is illustrated in Fig. S1 (Supporting Information). In this study, a NKN ceramic target with a composition in the morphotropic phase boundary region (Na_0.5_K_0.5_NbO_3_) was used to grow NKN nano-rods on a conductive 0.5 wt% Nb-doped SrTiO_3_ (Nb:STO) single-crystal (100) substrate from a laser ablated physical vapor. The working pressure and target-substrate distance were varied to optimize the growth conditions; however, the substrate temperature, laser energy, repetition rate, and number of laser pulses were maintained at a constant 700 °C, 1.6 J/cm^2^, 10 Hz, and 18000 shots, respectively. All of the NKN nano-rods in this study were grown under a working oxygen pressure of 200 mtorr, as shown in Fig. S2 (Supporting Information). Through this, the optimum target-substrate distance was determined to be 4 cm, as this provided the highest packing density of NKN nano-rods (see Fig.S3 in the Supporting information). Fig. 1a,b show the microstructure of NKN nano-rods grown under these optimum conditions, in which the NKN nano-rods were deposited on a NKN thin film and aligned along the vertical direction. The diameter and length of these nano-rods are approximately 60–90 and 500–600 nm, respectively, giving an aspect ratio of ~1:6.

The XRD analysis results shown in Fig. 1c reveal that both the NKN thin film and NKN nano-rods are highly oriented to the (001) plane that lies parallel to the (100) surface of the Nb:STO substrate, with the NKN nano-rods aligned along the [001] direction of the substrate. The out-of plane lattice parameter (*c*_NKN_) of the epitaxial NKN thin film and NKN nano-rods were calculated as 4.027 Å which is notably smaller than the lattice parameter of the Nb:STO substrate (*a*_Nb:STO_ = 3.914 Å). Given that the crystal structure of NKN with a morphotropic phase boundary (MPB) represents an orthorhombic symmetry (lattice parameter *a* = *c*)^28^, any residual in-plane compressive strain in the epitaxial NKN thin film is expected to be produced by a lattice mismatch in the in-plane spacing of the NKN thin film and Nb:STO substrate.

The cross-sectional TEM images in Fig. 1d demonstrate that a 160 nm-thick NKN thin film was grown on the Nb:STO substrate, with the inverse fast Fourier transform (IFFT) filtered HRTEM image of its interface with the Nb:STO substrate (Fig. 1e) confirming epitaxial growth and showing good agreement with the XRD data. The measured lattice parameters of the NKN thin film were determined as *c*_NKN_~4.031 Å and *a*_NKN_~3.973 Å, and are very different to those of bulk NKN (*a* = *c *~ 3.998 Å and *b ~ *3.935 Å)^28^. These results indicate that the lattice parameters of the epitaxial NKN thin film are distorted by residual in-plane compressive strain, which is clearly evident in the interface region (white dotted line). Fig. 1f shows a TEM image of a NKN nano-rod measuring 490 nm in length and 90 nm in width, with its accompanying IFFT filtered HRTEM image (Fig. 1g) revealing a vertical spacing of 3.992 Å along the growth direction. This value matches well with the lattice spacing of the (001) plane, and therefore clearly indicates that single-crystalline nano-rods were achieved through a relaxation of the in-plane residual compressive strain associated with the lattice mismatch between NKN and the Nb:STO substrate. To evaluate the composition of the NKN nano-rods, quantitative analysis was carried out by energy dispersive X-ray spectroscopy (EDS), the results of which are shown in Fig. S4 (a). Analysis of these EDS spectra revealed that the composition of the NKN nano-rod is very close to being in the MPB region (Na:K = 0.486:0.514).

The growth mechanism of the NKN thin film and nano-rods was also investigated through periodic observation of the cross-sectional microstructure. This found that an epitaxial NKN thin film is obtained after the first 4000 pulses, as shown in Fig. 2a,e, on which small islands of NKN are formed after 8000 pulses (Fig. 2b,f). With an increase to 12000 pulses, NKN nano-rods start to grow from these islands in a vertical direction (Fig. 2c,g), finally forming a fine and dense NKN nano-rod array after 18000 pulses (Fig. 2d,h). This particular growth behavior can be explained by the Stranski-Krastanov (SK) mode layer plus island growth mechanism, in which a transition from two-dimensional (2D) layer-by-layer to three dimensional (3D) island-based growth occurs at a critical thickness that is determined by the chemical and physical properties of the substrate and film^29^. Consequently, hetero-epitaxial film growth is dependent on the variation in specific interfacial free energy between the substrate and vapor (*γ*_sv_), film and substrate (*γ*_fs_), and film and vapor (*γ*_fv_). For SK mode growth, this relation between the interfacial free energies requires that^30^:
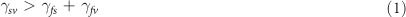


It is well known that the strain field induced by a lattice mismatch between a film and its substrate determines these interfacial free energies due to an accumulation of film-substrate strain energy in the growing film^30^. As such, the elastic relaxation energy increases with film thickness, leading to a coherent 3D island growth being preferred when the elastic energy exceeds that the free surface energy of the film^31^. Furthermore, although 3D islands are initially formed, subsequent growth of nano-rods helps to minimize the overall surface energy^27^.

Figure. S5 shows the XRD patterns and surface morphologies of NKN grown on pure SrTiO_3_ (STO) and Nb:STO substrates. In this, we see that since Nb ion substitution increases the lattice parameter, the lattice mismatch is much greater between NKN and a STO substrate. As a result, NKN grown on a STO substrate exhibits a much rougher thin-film surface morphology, and island growth dominated by a Volmer-Weber (VW) growth model. It is therefore apparent that the growth mode is very sensitive to the lattice mismatch between materials and substrates, with a certain strain required to ensure SK NKN nano-rod growth. However, other factors also need to be taken into consideration, with the specific interfacial free energies *γ*_sv_ and *γ*_fv_ being greatly influenced by the working pressure and energy of pulsed laser deposition. For instance, it is the working pressure that determines the density and size of the plume during SK growth transition, as evidenced by Fig. S2. Thus, under a high working pressure SK growth is suppressed (Fig.S2c and S2d), whereas a low working pressure induces SK growth (Fig.S2a and S2b). This variation in growth mode with working pressure is related to the change in distance between the plume and substrate (insets of Fig.S2), with SK growth more likely when the plume is closer. Varying the target-substrate (T-S) distance (see Supporting Information Fig. S3 and S6) revealed that the thickness of the nano-rods produced under a working pressure of 200 mtorr is increased by reducing the T-S distance; and in particular, the density of the nano-rods is significantly increased from 4 to 17 μm^-2^ when the T-S distance is changed from 5 to 4 cm, as shown in Fig. S7. Furthermore, in spite of the fact that only a small plume is produced under a high oxygen pressure of 400 mtorr, SK growth is still possible if the T-S distance is 3 cm (Fig. S6c). This highlights the importance of contact distance in determining the growth mode, and can be explained by the fact that laser-ablated materials in the plume lose kinetic energy through collisions with background gas molecules. This means that even though the plume size may be reduced, its interior still maintains a high density and high energetic condition^32^,^33^. A reduced contact distance therefore creates a physical vapor condition with a higher surface energy, which in turn changes the specific interfacial free energies *γ*_sv_ and *γ*_fv_ and allows for SK growth.

Using this relation between the kinetic energy of the laser ablated plume and the growth mechanism of NKN, a number of unique NKN nanostructures were obtained. Specifically, the kinetic energy of the plume can be easily controlled by changing the ablation laser energy, which also affects the formation of nuclei responsible for determining the crystalline size and density of the islands^30^. Fig. 2i–l shows the relation between the ablation laser energy (i.e., the kinetic energy of the plume) and the resulting surface morphology, in which we see that a low laser energy of 1 J/cm^2^ produces a 2D structured epitaxial NKN thin film. Increasing the ablation laser energy results in such structures as nano-islands (1.5 Jcm^2^), nano-rods (1.6 J/cm^2^) and nano-tapers (1.75 J/cm^2^), which confirms the feasibility of controlling the NKN nanostructure. Moreover, it provides an explanation as to how the vapor energy and nucleation rate during pulsed laser deposition affects the NKN nanostructure obtained.

The piezoelectric characteristics of the NKN nano-rods were evaluated by measuring the piezoelectric response phase and amplitude of the nano-rods and epitaxial thin films (as illustrated in Fig. 3a) using a 17 kHz 1 V sine modulation AC reading signal under an applied DC bias from −10 to 10 V. The piezoelectric response was collected from both the top of the nano-rods and the surface of the thin film in each sample, and from the resulting piezoelectric responses shown in Fig. 3b–d, it is evident that the phase signal of the NKN nano-rods exhibits a typical symmetric 180° domain switching behavior when the polarity of the DC bias is changed. Conversely, the NKN thin film shows a non-symmetric phase switching (Fig. 3b) that is known as an imprint phenomenon. The amplitude (Fig. 3c) and piezoelectric response (Fig. 3d) of the nano-rods is relatively high when compared with the NKN thin film. Furthermore, the film and NKN nano-rods both exhibit a non-symmetric piezoelectric response hysteresis that is associated with the electrostatic contribution when measuring PFM. In other words, since the electrostatic interaction usually occurs in the region surrounding the contact point, isolated NKN nano-rods show a smaller electrostatic contribution in non-symmetric behavior. A more detailed explanation of the electrostatic contribution to the piezoelectric response is provided in the Supporting Information, but the important point is that as a result of this phenomenon, relaxing the clamping effect through nano-structuring of the NKN helps to ensure a relatively high piezoelectric performance.

The *d*_33_, piezoelectric coefficient, of a clamped piezoelectric thin film tends to be degraded relative to its original piezoelectric properties, and so the effective piezoelectric coefficient is generally used to represent the piezoelectric performance of a clamped piezoelectric thin film. The effective piezoelectric coefficient (*d*_33eff_) of the clamped single crystal NKN was therefore calculated using Lefki and Dormans’s model^1^,^2^, in which *d*_33eff_ is related to the real piezoelectric coefficient (*d*_33_) as:
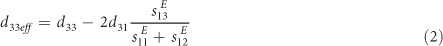
where, *s*_13_, *s*_12_, and *s*_11_ are the mechanical compliance of the piezoelectric film, and *d*_31_ is the transverse piezoelectric coefficient. The value of the constants for a NKN based single crystal poled along [001] were acquired from the literature^34^, and are detailed in Table S1 of the Supporting Information. The *d*_33eff_ value of a clamped single crystal of NKN was therefore determined to be 48.5 pm/V, a value which is significantly lower than that obtained with an unclamped single crystal (162 pm/V)^34^. In this study, to calculate *d*_33eff_ value of the NKN nano-rods and thin film from the piezoelectric response signal, an x-cut quartz crystal with top and bottom Pt electrodes was used as a reference specimen. To achieve this, its piezoelectric amplitude was measured between 0 and 5 V_rms_ of the AC reading signal. The slope of its linear amplitude versus the AC reading signal was obtained, which indicates the *d*_11_ value of the x-cut quartz (2.3 pm/V), and the effective piezoelectric coefficient values of the nano-rods and thin film were calculated from the piezoelectric response curve by comparison with the measured slope. To enhance accuracy, estimated experimental error was also included in the calculated data (see Fig. S8 in Supporting Information). The calculated effective piezoelectric coefficient curve is shown in Fig. S9, from which the average *d*_33eff_ values of the NKN thin film and NKN nano-rods were found to be 48 ± 5 and 97 ± 11 pm/V, respectively. Note that the effective piezoelectric coefficient of the NKN thin films is similar to that calculated for a clamped NKN single crystal, which indicates that the piezoelectric properties of the films are predominantly determined by the clamping effect. Whereas the NKN nano-rods exhibited higher effective piezoelectric coefficient value than the film. This means that the NKN nano-rods are relaxed from constraint by the clamping effect and restore their original piezoelectric coefficient value. However, this value is still less than that of a single crystal of NKN (~160 pC/N)^35^,^36^,^37^,^38^, because the piezoelectric coefficient of the NKN nano-rods is the sum of both the clamped thin film and unclamped nano-rods.

In summary, this study has shown that epitaxially grown single-crystalline NKN nano-rod arrays can be successfully obtained through conventional PLD methods. Growth under such conditions is considered to follow the Stranski-Krastanov (SK) mode layer plus island growth mechanism, and is therefore dependent on the physical vapor conditions. This means that careful control over the ablation laser energy, working pressure and T-S distance allows for the formation of different NKN nano-structures such as nano-island, nano-rods, and nano-tapers. The relaxation in substrate clamping that accompanies the formation of NKN nano-rods was shown to enhance the piezoelectric performance compared to a thin film, thus presenting an effective new method for fabricating environmentally friendly piezoelectric nano-structured materials for microelectronics. Moreover, the understanding gained of the growth mechanism represents a breakthrough in obtaining high-quality, single-crystalline and well-aligned complex metal oxide nano-structures.

## Methods

### Synthesis of NKN Nano-rod arrays

NKN nano-rods were grown on a 5 wt% Nb-doped SrTiO_3_ (Nb:STO) single-crystal substrate (100) using a conventional pulsed laser deposition (PLD) system with a KrF excimer laser (wavelength = 248 nm). A stoichiometric NKN ceramic pellet synthesized by solid state reaction was used as a target. To determine the optimum conditions, the substrate temperature, oxygen pressure, target-substrate distance, and repetition rate were maintained at 700 °C, 100~400 mtorr, 3–5 cm, and 10 Hz, respectively, during deposition. To ascertain the growth mechanism, the laser energy and number of laser pulses were varied between 1–1.75 J/cm^2^ and 4000–18000 shots, respectively.

### Characterization

The crystal quality of the NKN nano-rods was examined by X-ray diffraction (XRD: R_int_/D_max_ 2500, Rigaku Co., Japan) over a 2θ range of 20° to 60° using Cu-K_α_ radiation. The surface and cross-sectional microstructure of the nano-rods were observed using an environmental scanning electron microscope (ESEM: Philips FEI XL-30 FEG).To determine the growth orientation and crystal structure of the NKN nano-rods, bright field (BF) and high-resolution (HR) images were obtained by high-resolution transmission electron microscopy (HRTEM: FEI,Tecnai F20 G^2^).The effective piezoelectric coefficient (*d*_33eff_) of the NKN nano-rods was measured using a piezoelectric force microscope (PFM, Dimension 3100, Veeco Instruments, USA), with a lock-in amplifier (SR830, Stanford Research) utilized to simultaneously obtain the piezoresponse. All measurements were performed using a conductive Pt/Ir-coated Si-tip cantilever (PPP-NCHPt, Nanosensors) with a nominal spring constant of 43 N/m and a resonance frequency of 344 kHz. Piezoelectric response curves were acquired in contact mode using a typical modulation AC signal with a frequency of 17 kHz and an amplitude of 1 V. A piezoelectric response signal was collected from the top of the nano-rods and the surface of the thin film in each sample, from which piezoelectric responses curves depicting the phase, amplitude and piezoresponse were plotted using an average of values obtained from 9 nano-rods and 9 points on the film surface.

## Additional Information

**How to cite this article**: Kang, M.-G. *et al*. Enhanced piezoelectric properties of vertically aligned single-crystalline NKN nano-rod arrays. *Sci. Rep.* **5**, 10151; doi: 10.1038/srep10151 (2015).

## Supplementary Material

Supplementary Information

## Figures and Tables

**Figure 1:**
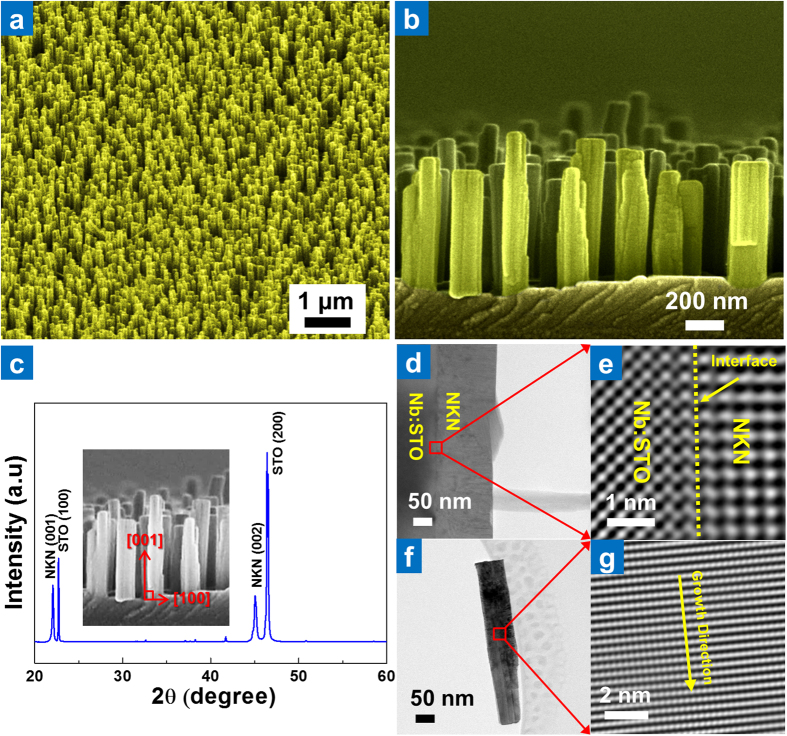
Structural characteristics of epitaxially grown NKN thin films and nano-rods. (**a**) Tilted-surface and (**b**) cross-sectional SEM images of vertically aligned NKN nano-rod arrays grown under optimized conditions (laser energy: 1.6 J/cm^2^, number of pulses: 18000, substrate temperature: 700 °C, working pressure: 200 mtorr, target-substrate distance: 4 cm).(**c**) XRD pattern of expitaxially grown NKN nano-rods/NKN thin film on a single-crystalline Nb:STO (100) substrate (inset shows the relation between the growth direction and the crystal orientation of the NKN nano-rods). (**d**) Cross-sectional TEM image of the epitaxially grown NKN thin film. (**e**) IFFT filtered HRTEM image of the interface between the NKN thin film and Nb:STO substrate. (**f**) TEM image of a NKN nano-rod. (**g**) IFFT filtered HRTEM image of a NKN nano-rod.

**Figure 2:**
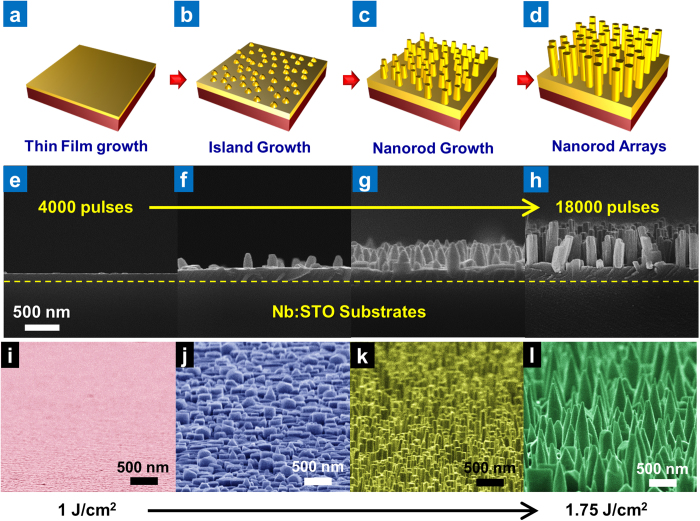
Physical growth of NKN nano-structures. Schematic diagram depicting the growth of NKN nano-rod arrays following (**a**) 4000, (**b**) 8000, (**c**) 12000, and (**d**) 18000 laser ablation pulses. (**e**)*-*(**h**) Cross-sectional SEM images of NKN nano-rod growth following (**e**) 4000, (**f**) 8000, (**g**) 12000, and (**h**) 18000 laser ablation pulses. (i)-(l) Tilted surface SEM image of NKN nanostructures grown using laser ablation energies of (**i**) 1, (**j**) 1.5, (**k**) 1.6, and (**l**) 1.75 J/cm^2^.

**Figure 3:**
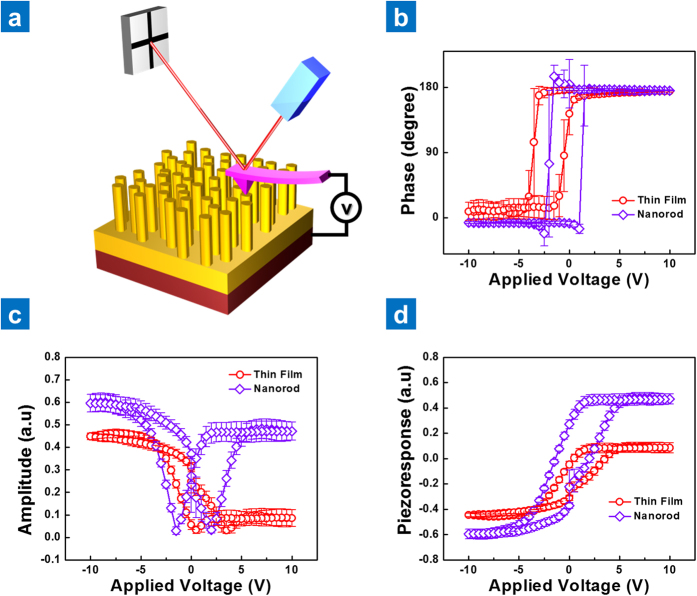
Piezoelectric characteristics of NKN nano-rods and NKN thin films. (**a**) Schematic showing PFM measurement of the piezoelectric characteristics of a single NKN nano-rod. DC bias induced (**b**) phase signal, (**c**) piezoelectric strain amplitude, and (**d**) *d*_33_-E curve of a single NKN nano-rod and NKN thin film.
