# Supplementary material for: Enhanced piezoelectric properties of vertically aligned single-crystalline NKN nano-rod arrays
Source: Sci Rep. 2015 May 8;5:10151. doi: 10.1038/srep10151 (PMC4650816; doi:10.1038/srep10151)
Supplement: Supplementary Information [file srep10151-s1.pdf]

Supplementary information

Enhanced piezoelectric properties of vertically aligned single-crystalline NKN nano-rod arrays

Min-Gyu Kang[1,3],Seung-Min Oh[1], Woo-Suk Jung[1], Hi Gyu Moon[1], Seung-Hyub Baek[1], Sahn Nahm[3], Seok-Jin Yoon[1]& Chong-Yun Kang[1,2,*]

[1]Electronic Materials Research Center, Korea Institute of Science and Technology, Seoul 136-791, Republic of Korea

[2]KU-KIST Graduate School of Converging Science and Technology, Korea University, 145, Anam-ro, Seongbuk-gu, Seoul, 136-701, Republic of Korea

[3]Department of Materials Science and Engineering, Korea University, 145, Anam-ro, Sungbuk-gu, Seoul 136-701, Republic of Korea

*e-mail: cykang@kist.re.kr

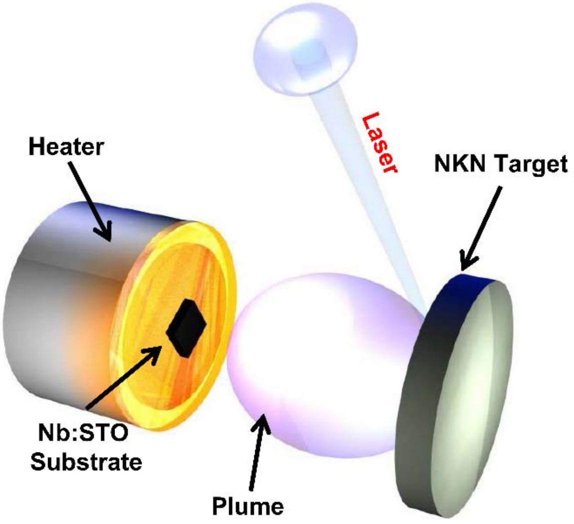

Supplementary Figure S1| A schematic illustration of the conventional pulsed laser deposition system used for the physical vapor growth of piezoelectric NKN nano-rod arrays.

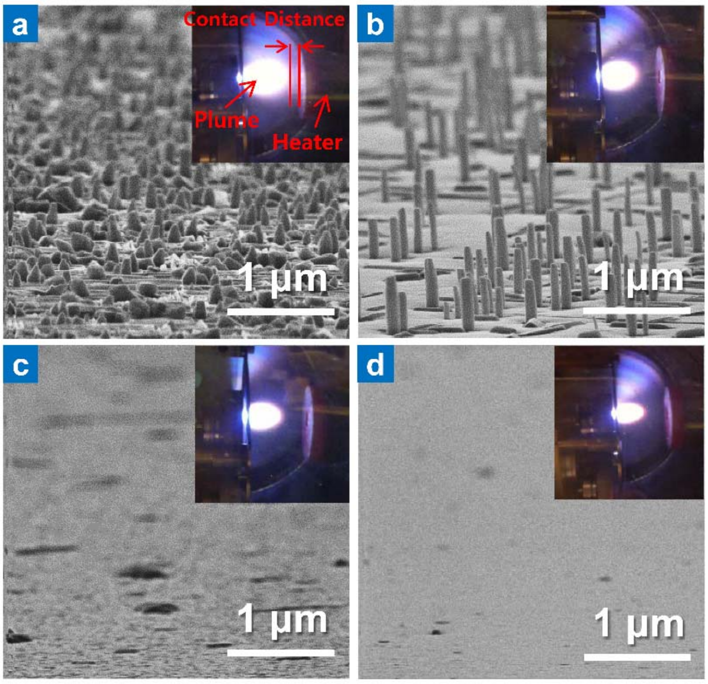

Supplementary Figure S2| SEM micrograph of NKN thin films and nano-rods fabricated on a Nb:STO single-crystal substrate under oxygen pressures of (a) 100, (b) 200, (c) 300, and (d) 400 mTorr.

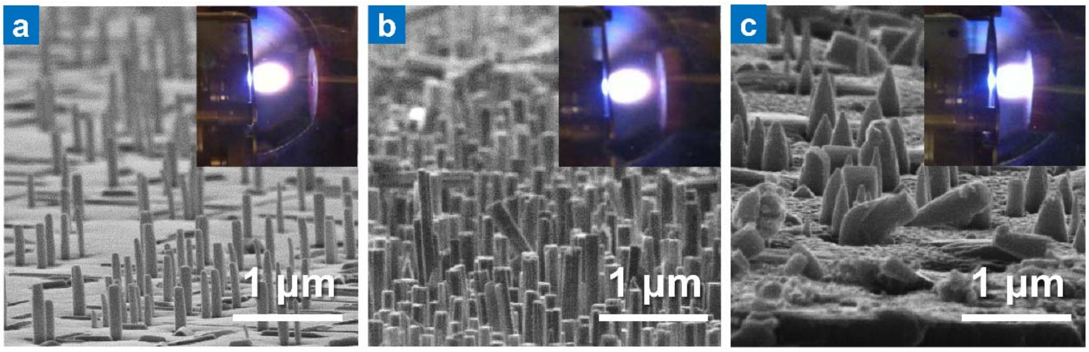

Supplementary Figure S3| SEM micrograph of NKN thin films and nano-rods deposited on a Nb:STO single-crystal substrate under 200 mTorr of oxygen pressure with T-S distances of (a) 5, (b) 4, and (c) 3 cm.

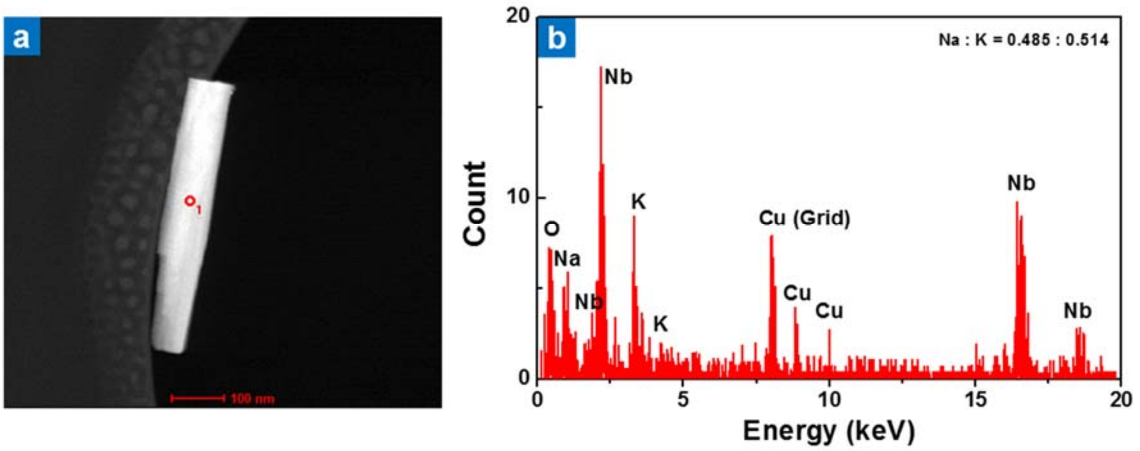

Supplementary Figure S4| EDS analysis of NKN nano-rod. (a) STEM image of the NKN nano-rod with measured point (red circle) (b) EDS spectra of NKN nano-rod.

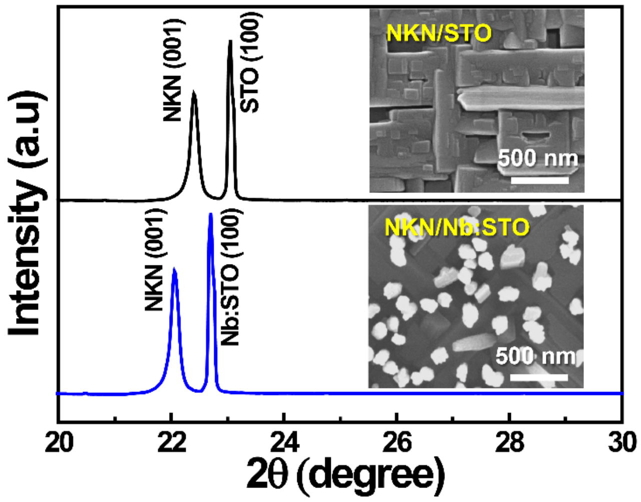

Supplementary Figure S5| XRD patterns and surface morphologies of NKN grown on pure STO or Nb-doped STO substrates.

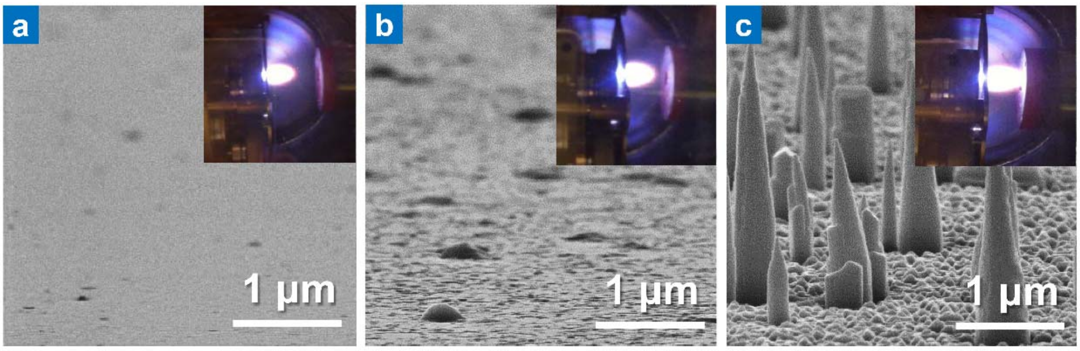

Supplementary Figure S6| SEM micrograph of NKN thin films and nano-rods deposited on a Nb:STO single-crystal substrate under 400 mTorr of oxygen pressure with T-S distances of (a) 5, (b) 4, and (c) 3 cm.

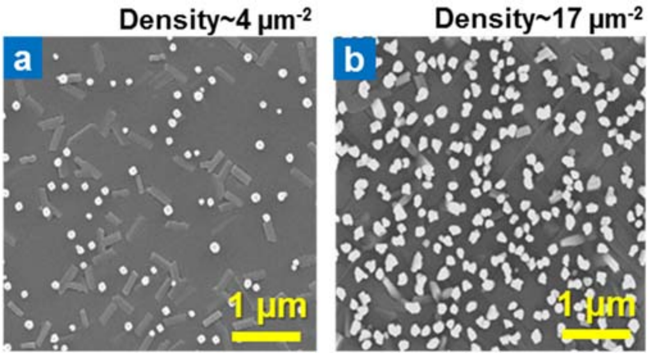

Supplementary Figure S7| Surface image of NKN nano-rod arrays deposited on a Nb:STO single-crystal substrate under 200 mTorr of oxygen pressure with T-S distances of (a) 5 and (b) 4 cm. The density of NKN nano-rods are 4 μm^{-2} and 17 μm^{-2} for T-S distance of 5 and 4 cm, respectively.

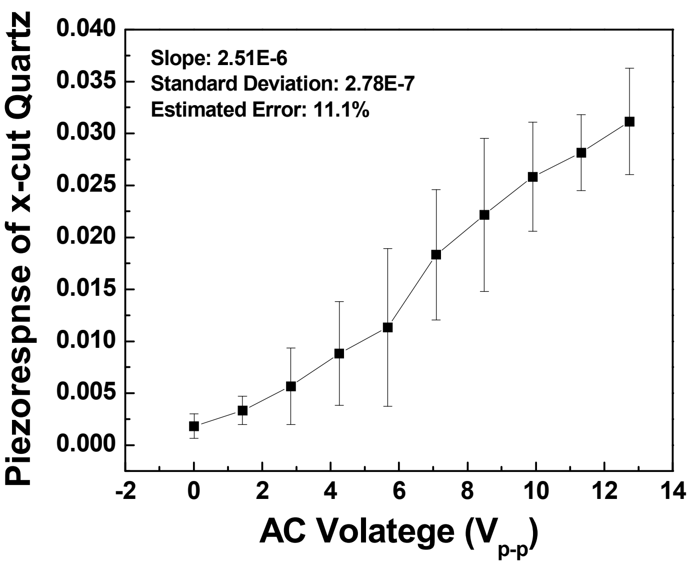

Supplementary Figure S8| Estimated error in slope of linear amplitude versus the AC reading signal plot of x-cut quartz.

A major error to determine d_{33eff} is occurred from system-inherent background when calibrating d_{33eff} value using x-cut quartz specimen, because the x-cut quart exhibits very small piezoresponse due to small d_{11} (2.3 pm/V)[1]. Therefore, we calculated estimated error in slope of linear amplitude versus the AC reading signal plot of x-cut quartz. We collected the piezoelectric amplitude signal from the 6 points of the x-cut quartz specimen as function of applied AC reading voltage and calculated standard deviation of the slopes of the each point as shown in Figure 1. We used the slope of 2.51E-6 to calculate d_{33eff} of the NKN nanorods and thin films, which has standard deviation of 2.78E-7. As a result, the estimated error was 11.1% and we applied this value in our PFM data to enhance accuracy.

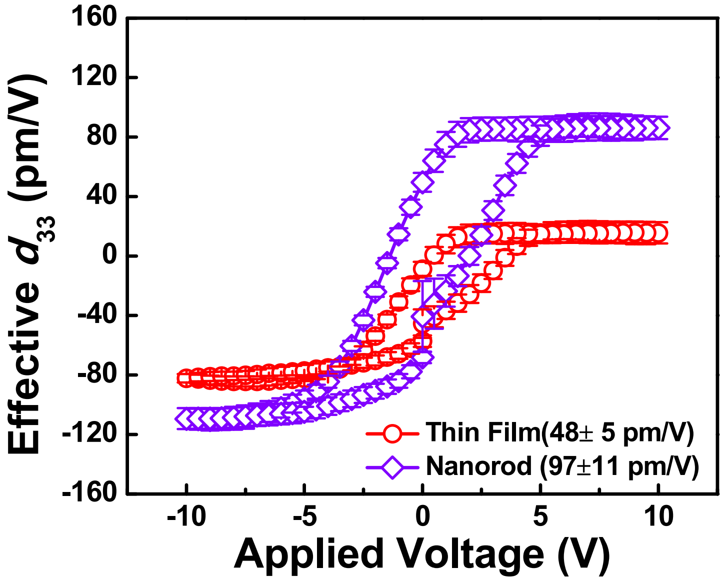

Supplementary Figure S9| Calculated effective piezoelectric coefficient curve of NKN nano-rods and thin films.

Supplementary Table S1| Piezoelectric constants of KNNT single crystal poled along [001][2]

Constants	s_{11} (10^{-12} m^2/N)	s_{12} (10^{-12} m^2/N)	s_{13} (10^{-12} m^2/N)	d_{31} (10^{-12} C/N)	d_{33} (10^{-12} C/N)
	11.9	-4.3	-5.6	-77	162

Non-symmetric behavior in piezoelectric response curve

From the principle of PFM, the asymmetry in the piezoresponse curve is usually observed due to electrostatic contribution between tip and surface of the specimen[3]. According to Sergei V. Kalinin, both electrostatic and electromechanical interactions contribute in PFM measurement and the piezoelectric response signal over c+ and c- domains can be written as[3]:

$$\mathrm{PR}(c+) = d_{33} + Q(V_{tip} - V_1) + F_{loc}(V_{tip} - V_1) + F_{nl}(V_{tip} - V_{av}) \quad (1)$$

$$\mathrm{PR}(c-) = -d_{33} + Q(V_{tip} - V_2) + F_{loc}(V_{tip} - V_2) + F_{nl}(V_{tip} - V_{av}) \quad (2)$$

where, V_{tip} is tip potential, V_{loc} is local potential below the tip apex, d_{eff} is effective electromechanical response of the surface, V_{av} is average surface potential below the cantilever, F_{loc} and F_{nl} are proportionality coefficients determined by tip-surface and cantilever surface capacitance gradients, tip-surface contact stiffness and spring constant of the cantilever. From this formula, the polarity of electrostatic contributed tip deflection is unchanged with changing polarization direction. The electrostatic contribution always causes attraction force between the tip and film surface. This force occurs deflection on c+ and c- domains with constructive and destructive contribution, respectively. In case of our study, the polarization polarity is changed by applied DC bias from 10 V to –10 V. Therefore, electrostatic contribution is expected as a main reason of the asymmetric piezoelectric response hysteresis. Moreover, stronger asymmetric behavior of piezoelectric response in the NKN thin film than NKN nano-rods can be also explained by this theory. This is because the electrostatic interaction usually occurs surrounding region from the contact point.

The real value of d_{33eff} and piezoelectric response in asymmetric piezoelectric response curve without electrostatic contribution can be obtained from,

$$[\mathrm{PR}(c+)-\mathrm{PR}(c-)] = d_{33} + Q(V_{tip} - V_1) + F_{loc}(V_{tip} - V_1) + F_{nl}(V_{tip} - V_{av}) + d_{33} - Q(V_{tip} - V_2) - F_{loc}(V_{tip} - V_2) - F_{nl}(V_{tip} - V_{av}) = 2d_{33}$$

Therefore, [PR(c+)-PR(c-)]/2 indicates the real effective d_{33} and piezoelectric response value. In this study we used d_{33eff} value in Fig. S9.

References

1. Soergel, E. Piezoresponse force microscopy (PFM). *J. Phys. D: Appl. Phys.* **44**, 464003 (2011).

2. Zheng, L. et al. Large size lead-free (Na,K)(Nb,Ta)O_3 piezoelectric single crystal: gorwth and full tensor properties. *Cryst. Eng. Comm*. **15**, 7718-7722 (2013).

3. Alexe, N. & Gruverman, A. *Nanoscale Characterisation of Ferroelectric Materials-Scanning Probe Microscopy Approach,* 12-15 (Springer, New York, 2004).
